# Characterization of *VuMATE1* Expression in Response to Iron Nutrition and Aluminum Stress Reveals Adaptation of Rice Bean (*Vigna umbellata*) to Acid Soils through *Cis* Regulation

**DOI:** 10.3389/fpls.2016.00511

**Published:** 2016-04-19

**Authors:** Meiya Liu, Jiameng Xu, Heqiang Lou, Wei Fan, Jianli Yang, Shaojian Zheng

**Affiliations:** ^1^State Key Laboratory of Plant Physiology and Biochemistry, College of Life Sciences, Zhejiang UniversityHangzhou, China; ^2^Key Laboratory of Tea Biology and Resources Utilization, Ministry of Agriculture, Tea Research Institute, Chinese Academy of Agricultural SciencesHangzhou, China; ^3^College of Resources and Environment, Yunnan Agricultural UniversityKunming, China

**Keywords:** acid soil, aluminum toxicity, *cis*-acting element, citrate secretion, evolution, Fe deficiency, transcriptional regulation

## Abstract

Rice bean (*Vigna umbellata*) *VuMATE1* appears to be constitutively expressed at vascular system but root apex, and Al stress extends its expression to root apex. Whether *VuMATE1* participates in both Al tolerance and Fe nutrition, and how *VuMATE1* expression is regulated is of great interest. In this study, the role of *VuMATE1* in Fe nutrition was characterized through *in planta* complementation assays. The transcriptional regulation of *VuMATE1* was investigated through promoter analysis and promoter-GUS reporter assays. The results showed that the expression of *VuMATE1* was regulated by Al stress but not Fe status. Complementation of *frd3-1* with *VuMATE1* under *VuMATE1* promoter could not restore phenotype, but restored with 35SCaMV promoter. Immunostaining of VuMATE1 revealed abnormal localization of VuMATE1 in vasculature. *In planta* GUS reporter assay identified Al-responsive *cis*-acting elements resided between -1228 and -574 bp. Promoter analysis revealed several *cis*-acting elements, but transcription is not simply regulated by one of these elements. We demonstrated that *cis* regulation of *VuMATE1* expression is involved in Al tolerance mechanism, while not involved in Fe nutrition. These results reveal the evolution of *VuMATE1* expression for better adaptation of rice bean to acid soils where Al stress imposed but Fe deficiency pressure released.

## Introduction

Multidrug and toxic compound extrusion (MATE) protein forms a large family of transporters in prokaryotes and eukaryotes where they perform a broad range of functions with diverse substrates (Omote et al., 2006). Some localize to the tonoplast where they transport secondary metabolites into the vacuole. For examples, anthoMATE in *Vitis vinifera* is an H^+^-dependent acylated anthocyanin transporter (Gomez et al., 2009) and the NtMATE1 and NtMATE2 proteins in *Nicotiana tabacum* are H^+^-dependent nicotine transporters (Shoji et al., 2009). EDS5 is a MATE protein in *Arabidopsis* exporting salicylic acid (SA) from the chloroplast to the cytoplasm (Serrano et al., 2013), while DTX50 functions as an abscisic acid (ABA) eﬄux transporter (Zhang et al., 2014).

A sub-group of MATE proteins in plants function as citrate transporters and perform at least two separate physiological roles. One role is involved in the translocation of iron (Fe) from the roots to the shoots, such as Ferric Reductase Deficient 3 (FRD3) in *Arabidopsis* (Durrett et al., 2007) and OsFRDL1 (for FRD3-like 1) in rice (*Oryza sativa*; Yokosho et al., 2009). These proteins are mainly expressed in the pericycle where they transport citrate into the xylem. Knockout mutations of these genes in *Arabidopsis* and rice result in Fe accumulation in the root vasculature. The second role for this sub-group of MATE proteins involves in Al^3+^ tolerance, such as HvAACT1 (Aluminum-activated citrate transporter 1) in barley (*Hordeum vulgare*; Furukawa et al., 2007) and SbMATE in sorghum (*Sorghum bicolor*; Magalhaes et al., 2007). These proteins are expressed in the root apices and they facilitate the Al^3+^-activated secretion of citrate which protects the growing root apices from Al^3+^ toxicity in acid soils. Therefore, the functions of MATE proteins that transport citrate depend on where they are expressed in the plant. Interestingly, recent evidence from rice and wheat (*Triticum aestivum*) indicates that a single citrate transporter can perform both physiological roles (Fujii et al., 2012; Tovkach et al., 2013). For instance, in Al^3+^-sensitive genotypes of barley, HvAACT1 is expressed in the vascular system of the root where it transports citrate into the xylem for efficient translocation of Fe to the shoots. However, in Al^3+^-tolerant genotypes of barley, a 1 kb insertion in 5′-UTR region of *HvAACT1* extends expression of this gene to the root apices that results in citrate secretion from these cells as well (Fujii et al., 2012). Similarly, *TaMATE1B* gene in the wheat is expressed in the vasculature of roots and induced by Fe deficiency. However, some Al^3+^-tolerant genotypes of wheat have a 11.1 kb transposon-like insertion in 25 bp upstream of the start codon of *TaMATE1B* which constitutively enhances its expression in root apices, increases citrate secretion from these cells and contributes to Al^3+^ tolerance (Tovkach et al., 2013). Thus, the physiological functions of these proteins have been extended through mutations which alter their tissue-specific expression pattern. These findings are consistent with the hypothesis that the original functions of these genes are altered or extended by mutations to the coding or regulatory regions of the gene (Ryan and Delhaize, 2010).

VuMATE1 from rice bean (*Vigna umbellata*) is another example of a MATE protein that transports citrate. It was first isolated from Al^3+^-stressed root tips and mediates Al^3+^-activated release of citrate from those cells (Yang et al., 2011). On the one hand, *VuMATE1* is not expressed in the root apices in the absence of Al stress, but induced by Al stress after 3 h of exposure (Liu et al., 2013). This raises the first question how the expression of *VuMATE1* was regulated under Al stress. Gene expression is regulated by different mechansims, such as chromatin condensation, DNA methylation, transcriptional initiaition, alternative splicing of RNA, mRNA stability and so on (Wray et al., 2003). In the case of *TaMATE1B* and *HvAACT1*, transposon-like element (TE) insertion in the promoter results in extention of expression into root apices and confers Al tolerancce phenotype. A miniature inverted repeat transposable element (MITE) insertion in the promoter region of *SbMATE* in sorghum was suggested to be involved in regulating the expression of this gene (Magalhaes et al., 2007). Whether similar mechanism is involved in *VuMATE1* expression regulation or not deserves to be investigated. On the other hand, *VuMATE1* seems to be constitutively expressed in the vascular system of mature roots cells (Liu et al., 2013). This raises the second question whether VuMATE1 plays roles in Fe nutrition as found for some other members of the family.

In this study, we first evaluated the contribution of *VuMATE1* to Fe nutrition using *in planta* complementation assays, and then explored the role of promoter (*cis*-regulatory sequences) in transcriptional regulation of *VuMATE1* expression in response to Al stress by means of GUS staining assays of *VuMATE1* promoter-GUS fusion transgenic lines. The results show that *VuMATE1* is expressed in epidermis and vasculature of mature roots but it is not involved in citrate release from roots of Fe deficient plants or in the translocation of Fe to the shoots. We further demonstrate that *cis*-regulation is involved in Al-induced expression of *VuMATE1*, and the *cis*-acting elements involved in root-tip-specific and Al-inducible expression of *VuMATE1* resided in promoter region between -1228 and -574 bp. We discuss these results with respect to the adaptation of rice bean to acid soils.

## Materials and Methods

### Isolation of *VuMATE1* Promoter

The promoter of *VuMATE1* was isolated from genomic libraries that have been constructed before (Liu et al., 2013). Nested PCR was performed using the outer/inner adaptor primer provided by the GenomeWalker^TM^ Universal Kit (Clontech, Mountain View, CA, USA) and two *VuMATE1* gene-specific primers (Supplementary Table S1). The amplified fragments were cloned into the pMD18-T vector (Takara, Dalian, China). The sequences that extends upstream of the cDNA clones were isolated as the 5′-upstream regions of the gene.

### Identification of Transcription Start Site

To determine the transcription start site (TSS) of *VuMATE1*, total RNA from Al-stressed root apices was isolated with an RNAprep pure Plant Kit (Tiangen, Beijing, China). 5′-RACE was performed using SMART^TM^ RACE cDNA Amplification Kit (Clontech). Gene-specific primers for 5′-RACE amplification are listed in Supplementary Table S1. Amplified cDNA fragments were cloned into the pMD18-T clone vector (Takara) and sequenced.

### Vector Construction and Genetic Transformation

A series of 5′ deleted promoters, -1720, -1228, -574, and -192 bp from the TSS of *VuMATE1*, were amplified by PCR method (Supplementary Figure S1). The primer sequences are shown in Supplementary Table S1. The amplified fragments were cloned into the pMD18-T vector (Takara) and their sequences were confirmed by DNA sequencing. The verified fragments were excised using *Kpn*I and *Nco*I sites at the 5′ ends of forward and reverse primers, respectively, and inserted at the corresponding sites of pCAMBIA1301. The plasmid containing -1720 bp construct was also cloned into pCAMBIA1302 at the *Kpn*I and *Nco*I sites creating an in-frame translational fusion to the GFP gene that was confirmed by DNA sequencing. These constructs was moved into *Agrobacterium* strain EHA105 and transformed into wild-type Col-0 plants (Clough and Bent, 1998).

For complementation assays, the *VuMATE1* coding sequence without stop codon was amplified via PCR method (Supplementary Table S1). The purified fragment was subcoloned into the constructed VuMATE1p*::GFP* plasmid between VuMATE1p and *GFP*. The generated plasmid was moved into *Agrobacterium* strain EHA105 and transformed into *frd3-1* homozygous lines.

For the construction of transgenic lines over-expressing VuMATE1, a previous constructed vector harboring 35S*::VuMATE1* was used to electroporate into EHA105 (Yang et al., 2011), and transformed into *frd3-1*.

For all transgenic plant lines, T1 seeds were collected from transgenic T0 plants, and were surface sterilized and planted on one-half-strength Murashige and Skoog medium supplemented with hygromycin. The resistant plants were transferred to soil and allowed to set seeds (T2). Transgenic lines that displayed a 3:1 ratio for hygromycin resistance in the T3 generation were selected for further analysis. All experiments were performed using plants corresponding to the T4 or T5 generation.

### Growth Conditions

For rice bean experiments, rice bean [*V. umbellata* (Thunb.) Ohwi and Ohashi] seeds were soaked in deionized water overnight, and germinated at 26°C in the dark. After germination, the seeds were transferred to a net floating on a 0.5 mM CaCl_2_ solution (pH 5.5). The solution was renewed daily. At day 3 after germination, seedlings were transferred to a 1.2-L plastic pot (four holes per pot, two seedlings for each hole) containing aerated one-fifth-strength Hoagland nutrient solution (pH 5.5). For Fe-deficiency experiment, plants were cultivated for 1 week in Fe sufficient conditions (1/5 Hoagland solution) and then transferred to the same nutrient solution with or without Fe-EDTA (20 μM) for 12 days. The treatment solution was renewed every 3 days.

For *Arabidopsis* experiments, seeds were surface-sterilized in 75% ethanol for 4 min, and subsequently rinsed thoroughly with sterile water. Seeds were stratified at 4°C for 2 to 4 days before being planted on Petri dishes with 1/5 Hoagland nutrient solution supplemented with 1 mM MES and 0.8% agar (pH 5.5). After 1 week, the seedlings with uniform size were selected to be transferred onto a net floating on one-fifth-strength Hoagland nutrient solution (pH 5.5). The solution was renewed every 3 days.

All of the experiments were carried out in an environmentally controlled growth room under 12 h 24°C : 12 h 22°C, light : dark, a light intensity of 200 μmol photons m^-2^ s^-1^ and 70% relative humidity.

### RT-PCR and qRT-PCR

Plant materials were ground in liquid nitrogen, and total RNA was extracted using an RNAprep pure Plant Kit (Tiangen). First-strand cDNAs were synthesized using a PrimeScript^TM^ RT-PCR Kit (Takara), and diluted to 100 ng μL^-1^. Semi-quantitative RT-PCR was performed with the diluted cDNA as template. Quantitative real-time PCR (qRT-PCR) was performed on a LightCycler480 machine (Roche Diagnostics, Indianapolis, IN, USA) using a SYBR PremixEx Taq kit (Takara). Primer pairs used in both RT- and qRT-PCR were listed in Supplementary Table S1. Three biological replicate RNA/cDNA samples were generated, and each cDNA sample was performed with triplicate technical replicates, from which the relative expression was calculated against that of the internal control gene *18S rRNA* using the formula 2^-ΔΔCp^.

### Collection and Analysis of Root Exudates

After treatments, the roots were briefly washed with 0.5 mM CaCl_2_ solution (pH 5.5), and then root exudates were collected in 0.5 mM CaCl_2_ solution (pH 4.5) either in the absence or presence of 25 μM Al for 6 h the collected root exudates were purified and concentrated according to Yang et al. (2006). The concentration of citrate was analyzed enzymatically (Delhaize et al., 1993).

### Immunostaining of VuMATE1

The synthetic peptide ATTDNNDIETGDEG-C (positions 173–186) was used to immunize rabbits to obtain antibodies against VuMATE1 (Genscript, Nanjing, China). One-week-old seedlings were used to immunolocalization of VuMATE1. The procedure was following previous report (Yamaji and Ma, 2007).

### Ferric Chelate Reductase Activity Measurement

Ferric chelate reductase (FCR) activity was determined according to the previous study (Yang et al., 2013). In brief, the whole excised root (c. 1.0 g) was placed in a tube filled with 50-ml of assay solution, which consisted of 0.5 mM CaSO_4_, 0.1 mM MES, 0.1 mM BPDS, and 100 μM Fe-EDTA at pH 5.5 adjusted with 1 M NaOH. The tubes were placed in a dark room at 25°C for 1 h, with periodic hand-swirling at 10-min intervals. The absorbance of the assay solutions was measured at 535 nm, and the concentration of Fe(II)[BPDS]_3_ was quantified using a standard curve.

### Chlorophyll Extraction and Quantification

After 3 weeks culture on hydroponics, newly expanded leaves were harvested. Chlorophyll was extracted in methanol and absorbance measured at 652, 665, and 750 nm. Total chlorophyll concentration was calculated as described (Porra et al., 1989).

### Microscopy

For GUS histochemical staining, seedlings (10-day-old) were incubated with the substrate 5-bromo-4-chloro-3-indolyl β-D-glucuronide as described (Jefferson et al., 1987). To localize Fe^3+^, seedlings after 3 weeks culture were vacuum-infiltrated with Perls stain solution [equal volumes of 4% (v/v) HCl and 4% (w/v) potassium ferrocyanide] for 30 min. Seedlings were then rinsed with water, observed, and photographed with a Nikon AZ100 microscope (Tokyo, Japan). The fluorescence of VuMATE1p::*GFP* in *Arabidopsis* was observed with a confocal laser scanning microscope (LSM710: Karl Zeiss, Jena, Germany). For imaging GFP, the 488 nm line of the Argon laser was used for excitation and emission was detected at 520 nm.

## Results

### *VuMATE1* Promoter Structure Analysis

To investigate regulatory mechanisms of *VuMATE1* expression, we analyzed the promoter sequence of *VuMATE1*. We obtained 2-kb DNA sequence upstream of *VuMATE1* translation start codon (ATG) (Supplementary Figure S1A). We next determined the potential transcription start site (TSS) of *VuMATE1* using 5′-RACE, and only one TSS (A, +1 position) in the *VuMATE1* promoter was identified (Supplementary Figure S1B). The TSS was located in the 3′ region of the putative TATA box (TATAA, -35/-30bp). There is an intron of 170 bp in length between TSS and translation start codon (Supplementary Figure S1A).

### Expression Patterns of *VuMATE1* under Fe Deficiency and Al Stress

We have previously demonstrated that the expression of *VuMATE1* is induced by Al stress in root tip region (Liu et al., 2013). However, tissue expression localization of *VuMATE1* in transgenic *Arabidopsis* plants carrying β*-glucuronidase* (GUS) reporter gene under control of *VuMATE1* promoter showed that *VuMATE1* is constitutively expressed at vasculature of maturation root zone (Liu et al., 2013). Here, we further examined the expression and localization of *VuMATE1 in vivo*. We transformed wild-type *Arabidopsis* (col-0) with a construct that harbors the *VuMATE1* promoter upstream of green fluorescent protein (VuMATE1p*::GFP*). GFP signal was not observed in root apex under normal growth conditions, but detectable in maturation root zone (Supplementary Figure S2A). Detailed analysis of the expression of VuMATE1p*::GFP* revealed that it was mainly expressed in epidermis and vasculature of maturation root zone (Supplementary Figures S2C,D). Exposure of roots to Al for 9 h resulted in significant increase of VuMATE1p*::GFP* expression in root apex (Supplementary Figure S2B), but had no effects in maturation root zone (Supplementary Figure S2D).

**FIGURE 1:**
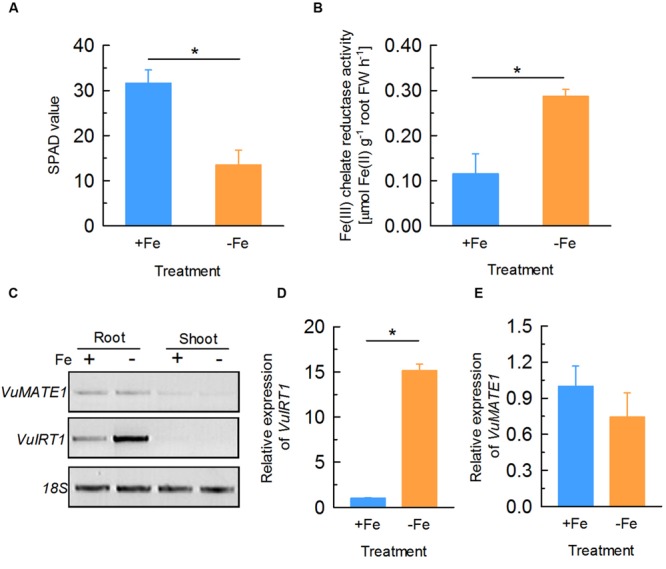
**Physiological and molecular responses of rice bean to Fe deficiency.** Seedlings of rice bean were subject to nutrient solution with 20 μM Fe (+Fe) or without (-Fe) for 12 days. **(A)** SPAD value of newly expanded leaves. Data are means ± SD (*n* = 8). **(B)** Ferric chelate reductase (FCR) activity of roots. Data are means ± SD (*n* = 4). **(C)** RT-PCR analysis of the expression of *VuMATE1* and *VuIRT1* in both roots and shoots. **(D)** Quantitative real-time PCR analysis of the expression of *VuIRT1* in roots. Data are means ± SD (*n* = 3). **(E)** Quantitative real-time PCR analysis of the expression of *VuMATE1* in roots. Data are means ± SD (*n* = 3). Asterisks represent significant differences between +Fe and -Fe treatment at *P* < 0.05.

To examine whether the expression of *VuMATE1* is regulated by Fe status, we analyzed the response of *VuMATE1* expression to Fe nutrition by RT- and qRT-PCR. After 12 days culture of rice bean seedlings in nutrient solution with (+Fe) or without Fe (-Fe), newly expanded leaves of -Fe plants displayed obvious chlorosis symptom (Supplementary Figure S3). SPAD values (indications of chlorophyll content) of newly expanded leaves decreased by ∼60% following this treatment compared to +Fe conditions (**Figure 1A**) and the ferric chelate reductase (FCR) activity in roots increased by almost threefold (**Figure 1B**), demonstrating that the plants were Fe-deficient after 12 days treatment. This is supported by RT-PCR and qRT-PCR analysis indicating that expression of *VuIRT1* (Iron Regulated Transporter 1), the ferrous Fe transporter, was fifteen times greater in the roots of -Fe plants compared to +Fe controls (**Figures 1C,D**). By contrast, *VuMATE1* expression in roots was unaffected by Fe status since expression levels were similar in the -Fe and +Fe plants (**Figure 1E**).

### *VuMATE1* cannot Complement the *frd3-1* Phenotype

**FIGURE 2:**
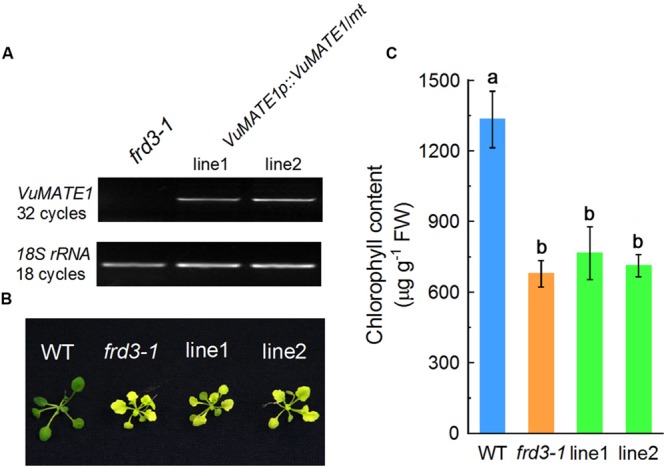
***In planta* complementation assay of *VuMATE1* in Fe nutrition in *Arabidopsis* mutant *frd3-1* driven by its native promoter (VuMATE1p*::VuMATE1/frd3-1*). (A)** RT-PCR characterization of *VuMATE1* expression in roots of two independent complemented lines (line 1 and line 2). **(B)** Phenotype analysis of leaf chlorosis in wild-type (WT), *frd3-1*, and two complemented lines. **(C)** Chlorophyll content of newly expanded leaves in wild-type (WT), *frd3-1*, and two complemented lines. Data are expressed as means ± SD (*n* = 4). Columns with different letters are significantly different at *P* < 0.05.

The localization of *VuMATE1* to the vascular system prompted us to investigate whether *VuMATE1* is also involved in Fe nutrition similar to *HvAACT1* in barley (Fujii et al., 2012) and *TaMATE1B* in wheat (Tovkach et al., 2013). We expressed *VuMATE1* using its native promoter in the *Arabidopsis* mutant *frd3-1* (VuMATE1p*::VuMATE1/frd3-1*), which is defective in Fe translocation (Green and Rogers, 2004), and two independent transgenic lines (line1 and line2) were used for further analysis. RT-PCR analysis indicated that *VuMATE1* was expressed in both transgenic lines but not in *frd3-1* mutant (**Figure 2A**). When grown in one-fifth-strength Hoagland nutrient solution, newly expanded leaves of the *frd3-1* mutant lines exhibited severe chlorosis (**Figure 2B**), which is in accordance with lower chlorophyll levels (**Figure 2C**). Perls blue staining demonstrated that significantly more Fe was accumulated in the root vasculature of *frd3-1* than WT plants (Supplementary Figure S4), which is consistent with previous descriptions of this mutant (Durrett et al., 2007). The *frd3-1* lines transformed with VuMATE1p*::VuMATE1* showed these same general symptoms (**Figure 2B**). Chlorophyll content of the newly expanded leaves in *frd3-1* and the two independent complementation lines was approximately half of WT levels (**Figure 2C**). Perls blue staining of roots showed similarly high Fe precipitation in the vasculature of the complementation lines (Supplementary Figure S4), indicating that Fe translocation to the shoots was reduced in all the lines. These results indicate that *VuMATE1* expression driven by its native promoter could not complement the mutant phenotype of *frd3-1*.

**FIGURE 3:**
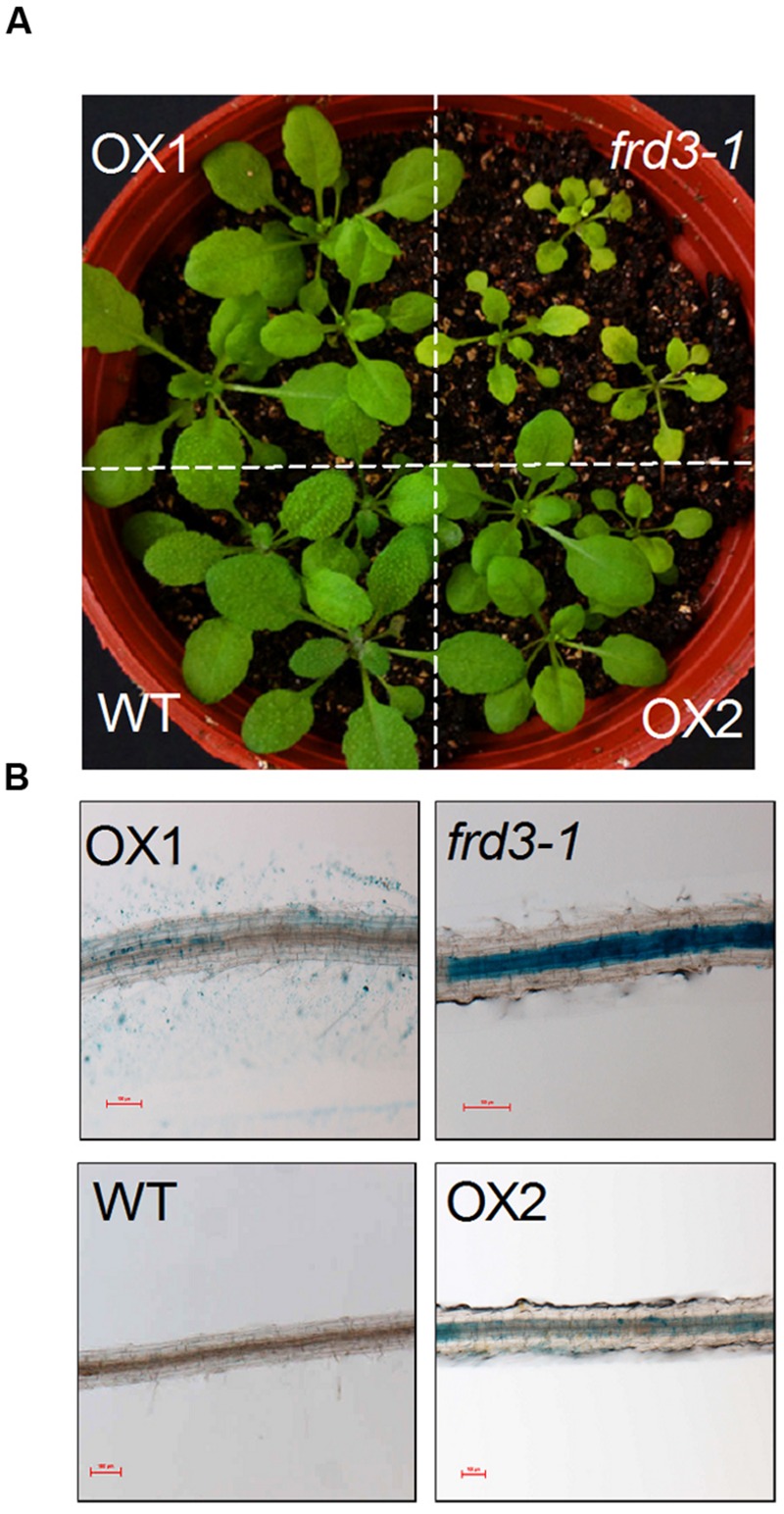
***In planta* complementation assay of *VuMATE1* in Fe nutrition in *Arabidopsis* mutant *frd3-1* driven by 35S CaMV promoter. (A)** Phenotype analysis of leaf chlorosis in WT, *frd3-1*, and two transgenic lines overexpressing *VuMATE1* (OX1 and OX2). **(B)** Root ferric precipitation of WT, *frd3-1*, and two transgenic lines overexpressing *VuMATE1* (OX1 and OX2). Bar, 100 μm.

We have previously demonstrated that VuMATE1 is a plasma membrane-localized citrate-permeable transporter protein (Yang et al., 2011). Thus, the inability of *VuMATE1* to restore *frd3-1* phenotype with respect to Fe nutrition suggests that the expression pattern but not gene function is responsible for the loss of its role in Fe nutrition. To test this hypothesis, we introduced *VuMATE1* using 35S CaMV promoter into the *frd3-1* mutant (35S*::VuMATE1/frd3-1*). The chlorosis was greatly, albeit not completely, restored in two independent transgenic lines, OX1 and OX2 (**Figure 3A**). In addition, Perls blue staining result also showed that the accumulation of Fe in root vasculature was decreased dramatically in comparison to *frd3-1* mutant (**Figure 3B**).

### Cell-Specificity of Localization of VuMATE1

**FIGURE 4:**
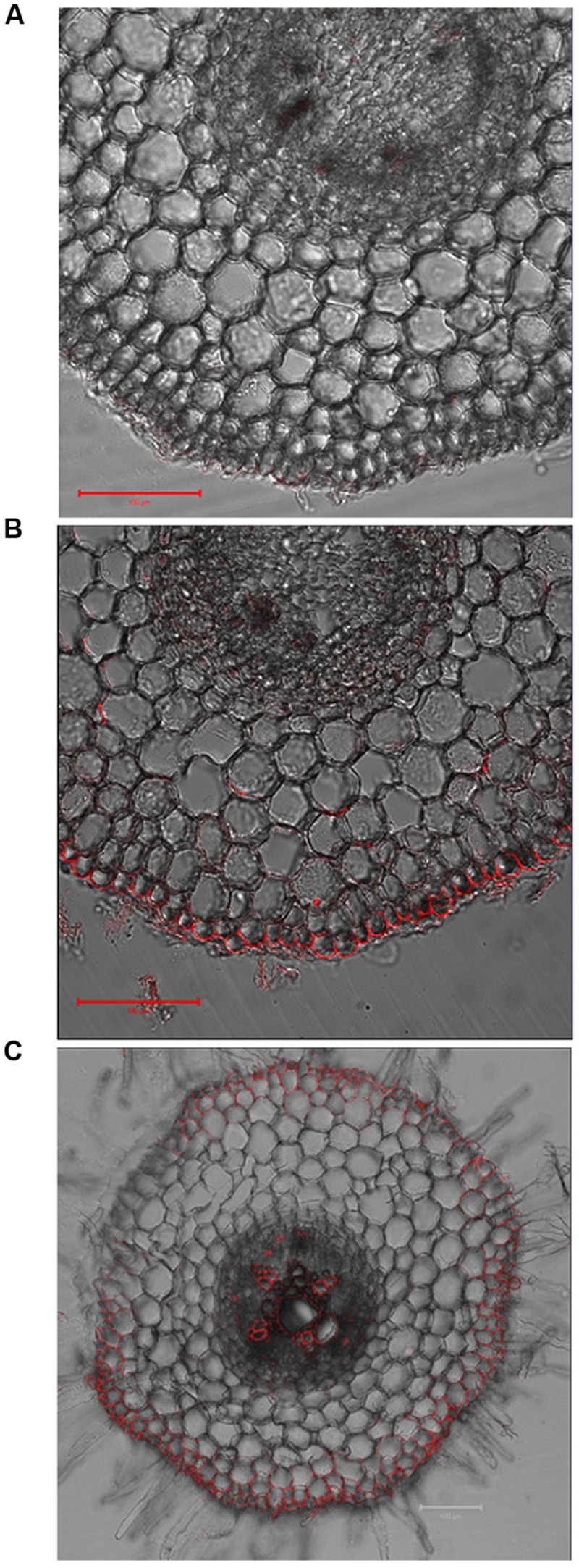
**Cell-specificity of localization of VuMATE1 in roots.** Immunostaining with anti-VuMATE1 antibody is shown in the root apex either before **(A)** or after Al stress for 9 h **(B)**, and maturation root zone **(C)**. Bar, 100 μm.

To further investigate why *VuMATE1* expression under the control of its native promoter could not complement the *frd3-1* mutant, we examined the cell-specificity of localization of VuMATE1 with a polyclonal antibody. In root apex, no fluorescent signal was observed (**Figure 4A**). However, fluorescence signal was detected in the epidermis of root apex after 9 h exposure to 25 μM Al (**Figure 4B**). Moreover, VuMATE1 is localized on the plasma membrane of the distal side of epidermis cell (**Figure 4B**). Being mainly localized to cells near xylem vessels, and the epidermis in maturation root zone, VuMATE1 could not be detected in the pericycle or in cells internal to the pericycle (**Figure 4C**).

### Effect of Fe Supply on Citrate Secretion

**FIGURE 5:**
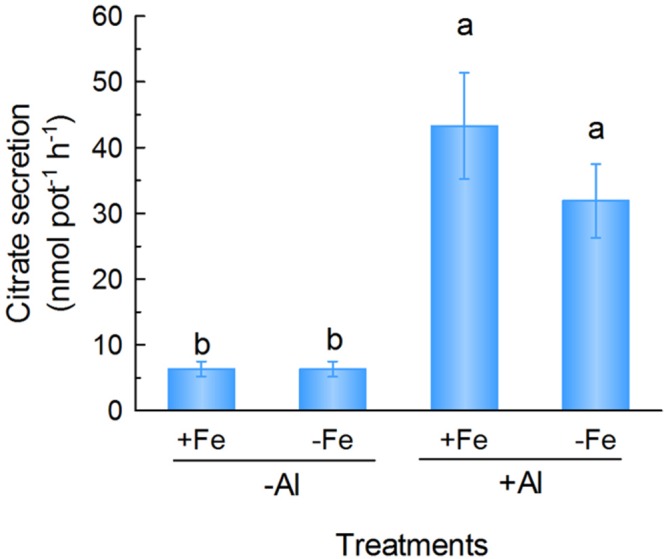
**Citrate secretion from rice bean roots in response to Fe deficiency and Al stress.** One-week-old seedlings were subject to nutrient solution with 20 μM Fe (+Fe) or without (-Fe) for 12 days after treatment, root exudates were collected in 0.5 mM CaCl_2_ solution (pH 4.5) in the absence (-Al) or presence of 25 μM Al (+Al) for 6 h. Data are expressed as means ± SD (*n* = 4). Columns with different letters indicate significant difference at *P* < 0.05.

Citrate anion secretion from roots can also complex with Fe in the rhizosphere to improve its mobilization and uptake by roots (Jones et al., 1996). The localization of VuMATE1 in the epidermis cells of the root led us to investigate its possible role in mobilizing Fe in the rhizosphere through enhanced citrate secretion. Therefore, we analyzed citrate secretion from whole roots under either +Fe or -Fe conditions and found that Fe deficiency did not increase citrate secretion. However, Al treatment significantly increased citrate secretion regardless of Fe supply (**Figure 5**).

### Promoter Analysis to Characterize *VuMATE1* Expression

**FIGURE 6:**
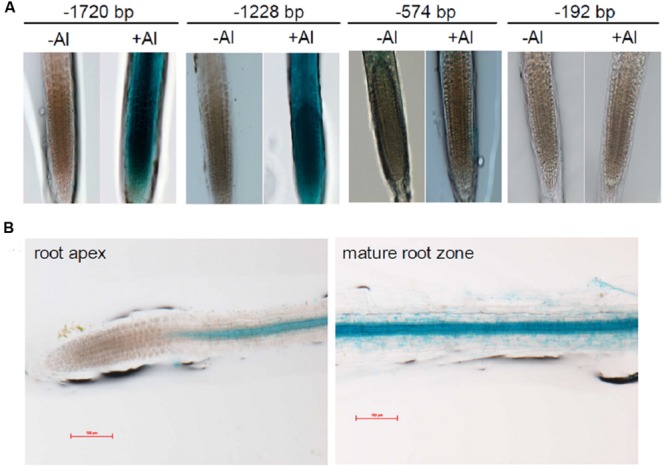
**Histochemical analysis of GUS expression in transgenic *Arabidopsis* lines driven by different 5′ deletion sequence of *VuMATE1* promoter. (A)** Induction of *VuMATE1* by Al in transgenic *Arabidopsis* carrying VuMATE1p*::GUS* constructs. The representative images show GUS staining in roots after 9 h of exposure to 0 (-Al) or 1 μM Al (+Al) activity in nutrient solution. **(B)** GUS expression in root apex and maturation root zone of transgenic *Arabidopsis* carrying the shortest promoter of *VuMATE1* (-192 bp*::GUS*). At least two independent transgenic lines were used to analyze GUS expression. Bar, 100 μm.

To characterize in detail the regulation of *VuMATE1* transcription by Al stress and Fe status, we analyzed GUS expression in VuMATE1p*::GUS* transgenic lines carrying different lengths of the 5′ region of the *VuMATE1* promoter. In the absence of Al stress, GUS staining was not observed in the root apex of all promoter-GUS reporter transgenic lines carrying different 5′ deletion promoters (**Figure 6A**). However, after 9-h exposure to Al stress, promoter-GUS reporter lines, -1720 and -1228 bp*::GUS*, exhibited Al-inducible and root apex staining of GUS activity, whereas GUS staining was not observed in -574 and -192 bp*::GUS* transgenic lines (**Figure 6A**). This result indicated that *cis*-acting elements responsible for root-tip specific and Al-inducible expression resided in *VuMATE1* promoter sequence between -1228 and -574 bp. On the other hand, all promoter-GUS reporter transgenic lines examined showed constitutive expression in maturation root zone, even in the shortest -192 bp*::GUS* transgenic line (**Figure 6B**).

To find *cis*-acting elements in the promoter, we first searched the *VuMATE1* promoter sequence between -1228 and -574 bp using the PLACE and PlantCARE databases (Higo et al., 1999; Lescot et al., 2002). As shown in **Table 1**, both databases predicted *cis*-acting elements related to drought-inducible element (MBS), ABA response element (ABRE), wounding and pathogen responsive element (W box), and SA response element (TCA). We then searched three reported Al-responsive *cis*-acting elements, i.e., GGN(T/g/a/C)V(C/A/g)S(C/G)T of STOP1 ortholog, ART1 (Tsutsui et al., 2011), GGCCCA(T/A) of ASR5 (Arenhart et al., 2014), and CGCG box of CALMODULIN-BINDING TRANSCRITPION ACTIVATOR (CAMTA; Tokizawa et al., 2015). Only a single *cis*-acting element of ART1 was found to be located at -629 bp, whereas none of both ASR5 and CAMTA was observed (**Table 1**).

**Table 1:** The list of *cis*-acting elements of the *VuMATE1* promoter sequence between -1228 and -574 bp.

*cis*-acting element	Motif	Position	Organism	Function
ABRE	TGCACGTAT	-610	*Arabidopsis thaliana*	*cis*-acting element involved in the abscisic acid (ABA) responsiveness
MBS	CGGTCA	-808	*Zea mays*	MYB binding site involved in drought-inducibility
	CAACTG	-586	*Arabidopsis thaliana*	
W box	TTGACC	-1009	*Arabidopsis thaliana*	Elicitation; wounding and pathogen responsiveness. Binds WRKY type transcription factors
TCA	TGTTCTTCTC	-1224	*Brassica oleracea*	Salicylic acid response element
ART1	GGNVST	-629	*Oryza sativa*	Al stress responsiveness

**FIGURE 7:**
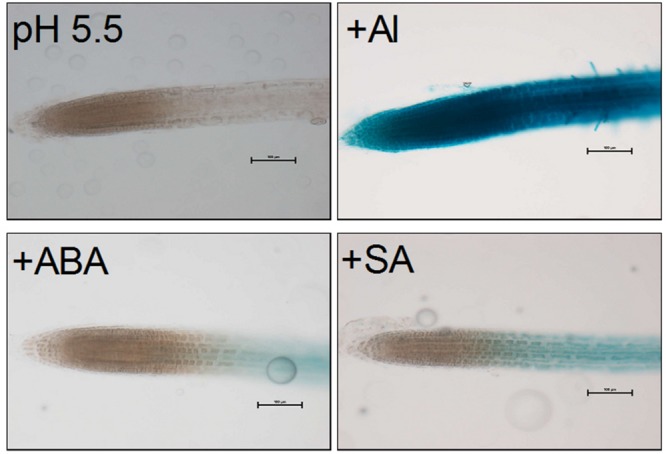
**VuMATE1p*::GUS* expression analysis in transgenic *Arabidopsis* plants.** Transgenic seedlings were exposed to 10 μM Al, 10 μM ABA, or 10 μM SA for 9 h. Activation of the *VuMATE1* promoter was observed by GUS staining (blue). At least two independent transgenic lines were used to analyze GUS expression. Bars, 100 μm.

In order to examine whether phytohormones, ABA and SA, are involved in the transcriptional regulation of *VuMATE1*, we compared GUS activities among different treatments, i.e., ABA (10 μM; pH 5.5), SA (10 μM; pH 5.5), or Al (10 μM; pH 5.5). Previous study showed that apart from Al, low pH (5.0) also affect *VuMATE1* expression in GUS transgenic lines (Fan et al., 2015). Therefore, in order to clarify the regulatory role of hormones, we used the pH5.5 as normal growth conditions to eliminate the impact of pH. As shown in **Figure 7**, under normal growth conditions (pH 5.5), GUS activity was undetectable in the entire root apex region. Both ABA and SA could slightly induce GUS activity restricted to elongation zone. Addition of Al resulted in the strong induction of GUS activity at entire root apex region.

## Discussion

In the present study, we demonstrated that *cis* regulation is involved in the transcriptional regulation of *VuMATE1* expression in adaptation to acid soils. *In planta* GUS expression analysis indicated that *cis*-acting motifs responsible for Al-inducible expression reside between -1228 and -574 bp of *VuMATE1* promoter (**Figure 6**). Several lines of evidence suggest that the acquisition of Al tolerance mechanism can occur through mutations that modify the expression patterns of a single gene. For example, in wheat, the number of tandemly repeated elements of 33–803 bp in length located upstream of *TaALMT1* coding region was associated with higher levels of *TaALMT1* expression (Sasaki et al., 2006; Ryan et al., 2010). Interestingly, these tandemly repeated elements are absent in the *AetALMT1* which is the ortholog of *TaALMT1* from *Aegilops tauschii*, the donor of D genome in hexaploid wheat (Ryan et al., 2010). It was speculated that these tandemly-repeated elements provided some advantage to hexaploid wheat as individuals spread to more acid soils. In Al-tolerant genotypes of barley, a 1-kb insertion upstream of the *HvAACT1* coding region extends the expression of this gene into the root apex (Fujii et al., 2012). Similarly the higher expression level of *TaMATE1B* in several Brazilian wheat lines was found to be associated with the presence of a Sukkula-like transposable element in its promoter region as well (Tovkach et al., 2013). Apparently, the acquisition of Al tolerance mechanism in rice bean is different from these species, since no insertion segments were observed in the *VuMATE1* promoter.

Recently, adaptation of *Holcus lanatus* to acid soils was achieved by increasing the number of *cis*-acting elements for the transcription factor, ART1, in the promoter of *HlALMT1* (Chen et al., 2013). The expression of *Arabidopsis AtALMT1* is also largely attributed to the interaction between AtSTOP1 and its *cis*-acting element. However, the *cis*-acting elements and trans-regulatory factors involved in *VuMATE1* expression seem to be different from these genes. We have previously demonstrated that an inducible C_2_H_2_-type zinc finger transcription factor, VuSTOP1, can bind to *VuMATE1* promoter region between -1228 and -920 bp, but not to the region between -820 and -555 bp where a putative *cis*-acting motif of ART1 resides (**Table 1**; Fan et al., 2015). Furthermore, *in planta* complementation of *Atstop1* mutant with *VuSTOP1* demonstrated that the expression of *AtMATE* was only slightly restored in the complemented lines (Fan et al., 2015). Thus, it is very likely that transcription factors other than VuSTOP1 are involved in the transcriptional regulation of *VuMATE1* under Al stress. Recently, a series of *cis*-acting elements and transcription factors responsible for the expression regulation of *AtALMT1* were identified by integrating bioinformatics and molecular biological approaches (Tokizawa et al., 2015). Thus, it is possible that multiple mechanisms are also involved in *VuMATE1* expression regulation, which needs further investigation.

We identified a putative W-box *cis*-acting motif (TTGACC) in the position of -1009 bp of *VuMATE1* promoter (**Table 1**), which is potentially responsible for the interaction with WRKY-type transcription factors. Recently, Ding et al. (2013) reported that WRKY46 functions as a repressor of *AtALMT1*, whereas WRKY46 itself is repressive to Al stress. Given that WRKY-type transcription factors were involved in the transcriptional regulation of *VuMATE1* expression in rice bean, they will perform as a transcriptional activator instead of repressor. This is evidenced by GUS expression in VuMATE1p*::GUS* transgenic lines carrying different lengths of the 5′ region of the *VuMATE1* promoter. In both -579 and -194 bp*::GUS* lines, GUS activity was undetectable either in the absence or presence of Al stress (**Figure 6A**), indicating it is not repressors but activators to be mainly involved in *VuMATE1* expression. Furthermore, pretreatment with CHX, a protein translation inhibitor, resulted in the significantly inhibition of *VuMATE1* expression (Liu et al., 2013; Fan et al., 2015), confirming that repressor is not involved in the transcriptional regulation of *VuMATE1* expression in response to Al stress.

Phytohormones are important signal inducers that participate extensively in plant response to environmental stresses (Sun et al., 2010; Tian et al., 2014; Yang et al., 2014). In soybean, ABA was reported to enhance citrate secretion both in the absence and presence of Al stress (Shen et al., 2004). Al stress resulted in the accumulation of SA, and exogenously application of SA could in turn improve Al tolerance through modulation of citrate secretion in *Cassia tora* (Yang et al., 2003). In this present study, we identified both potential ABA-responsive and SA-responsive *cis*-acting elements in the promoter of *VuMATE1* (**Table 1**), suggesting possible involvement of these phytohormones signaling in Al-regulated *VuMATE1* expression. However, we found that phytohormones (both ABA and SA) are not the major factors responsible for Al-induced *VuMATE1* expression in rice bean root tip, although they induced GUS activity. *In planta* GUS expression analysis revealed that there were differences in the intensity and tissue localization of the GUS staining between phytohormones (ABA and SA) and Al treatments (**Figure 7**). Recently, it was demonstrated that several signal inducers, especially IAA and ABA, can trigger *AtALMT1* expression in *Arabidopsis* (Kobayashi et al., 2013). However, the induction of *AtALMT1* expression in response to Al stress is independent of IAA and ABA signaling, which in combination with our present observation provides evidence that Al-induced organic acid secretion seems to be independent of signaling from phytohormones.

We found that in control conditions (-Al) the expression of *VuMATE1* was absent in the root apex, but constitutively expressed in root hairs, epidermis and vasculature of mature roots (Supplementary Figure S2; **Figure 6**). This pattern of expression suggested that VuMATE1 could be involved in aspects of Fe nutrition in rice bean. This is based on previous reports that citrate secretion from roots is associated with Fe mobilization from the rhizosphere in chickpea (*Cicer arietinum*; Ohwaki and Sugahara, 1997) and maize (*Zea mays*) (Carvalhais et al., 2011). Furthermore, other citrate-transporting MATE proteins such as HvAACT1 in barley (Fujii et al., 2012), TaMATE1B in wheat (Tovkach et al., 2013), FRD3 in *Arabidopsis* (Rogers and Guerinot, 2002) are induced by Fe deficiency and likely responsible for Fe translocation to the shoots by loading citrate into xylem. However, here we demonstrated that VuMATE1 was not involved in Fe nutrition in rice bean. This conclusion is supported by the following pieces of evidence.

First, *VuMATE1* expression was not affected by Fe deficiency (**Figure 1E**). There are two potential reasons responsible for the inability of responsiveness of *VuMATE1* expression to Fe nutritional status. One is that there are no Fe-deficiency-responsive components regulating *VuMATE1* expression in rice bean. However, since both *VuIRT1* expression and FCR activity was induced by Fe deficiency (**Figures 1B,D**), Fe-deficiency-responses are clearly operating in rice bean. The other reason is that there are either no Fe-deficiency-inducible *cis*-elements in the promoter of *VuMATE1*, or that such *cis*-elements were originally present but have been lost or mutated during the adaptation of rice bean to acid soils. Acid soils typically have toxic concentrations of Al but they rarely induce Fe deficiency in plants because the low pH can maintain a higher concentration of soluble Fe. Therefore there is less requirements to mobilize Fe from acid soil through the secretion of organic anions. In line with this result, there is only a trace amount of citrate secreted into growth medium and the secretion rate was unaffected by the onset of Fe deficiency (**Figure 5**). Thus, it is possible that as these plants adapted to acid soils they lost the *cis*-elements responsible for iron-deficiency-inducible expression of *VuMATE1* but maintained the induction of expression by Al. Such a change could confer an advantage to rice bean because in addition to improving Al tolerance, it would minimize unnecessary carbon loss in conditions when it would not be beneficial. This is supported by the finding that transgenic tomato lines that express *VuMATE1* under control of constitutive 35S CaMV promoter exhibited constitutive citrate eﬄux (Yang et al., 2011).

The second reason for concluding that VuMATE1 is not involved in Fe nutrition is that VuMATE1 does not appear to translocate Fe from the roots to the shoots. This was supported by the finding that transgenic *Arabidopsis* lines expressing *VuMATE1* under the control of its native promoter could not rescue the phenotype of *frd3-1* mutant which is defective in Fe translocation (**Figure 2**). There are two possible reasons for the ineffectiveness of VuMATE1 in Fe translocation. One is that the expression level of *VuMATE1* was too low to exhibit a sufficient phenotype (**Figure 1C**). However, *FRD3* expression in wild-type *Arabidopsis* is also at the limits of detection yet this gene was able to complement the *frd3-1* mutant (Green and Rogers, 2004). Thus, the low level of *VuMATE1* expression may not be the main reason for its inability to translocate Fe. An alternative possibility is that the tissue-specific localization of VuMATE1 is unsuited for Fe translocation. Both *FRD3* in *Arabidopsis* and *OsFRDL1* in rice have been demonstrated to be localized mainly in the pericycle (Green and Rogers, 2004; Yokosho et al., 2009). However, the expression of *VuMATE1* was absent in pericycle and cells immediately internal to pericycle (**Figure 4**). Although it is still not clear how critical localization to the pericycle is for Fe translocation, the restoration of phenotype by *VuMATE1* under 35S CaMV promoter (**Figure 3**) suggests that this difference in localization contribute to the ineffectiveness of VuMATE1 in helping Fe move to the shoots.

Why then does VuMATE1 appear to lack a role in Fe nutrition whereas other citrate transporters from the MATE appear to be involved in Fe nutrition as well as Al tolerance? The explanation might be found in the very different origins of rice bean compared to barley and wheat. For example, barley and wheat arose and were originally cultivated in regions with calcareous soils where Fe deficiency is a major constraint to growth (von Bothmer et al., 2003; Ryan et al., 2010). Therefore, both species evolved efficient mechanisms for accessing and taking up Fe from these soils. The adaptation of these species to more acidic soil may have been long enough to evolve Al tolerance but not to lose their efficient mechanisms for acquiring Fe. Indeed, a recent report concluded that selection pressure for greater Al tolerance in natural populations of *H. lanatus* required only 150 years for Al tolerant alleles of *HlALMT1* to become more frequent in the surviving population (Chen et al., 2013). By contrast, rice bean was originally cultivated on acid soils where Al toxicity poses a stronger selection pressure than Fe deficiency (Yang et al., 2006). We hypothesized that the Fe-deficiency-responsive *cis*-acting element does not evolve for *VuMATE1* or such element has lost during long adaptation process to acid soils. The GUS reporter transgenic line carrying the shortest promoter exhibited the same expression patterns to others carrying longer promoter in term of Fe nutrition status provided the circumstantial evidence to support our hypothesis (**Figure 6B**). However, the evolution of root tip-specific and Al-inducible *cis*-elements in rice bean not only alleviates Al toxicity but also prevents excessive loss of fixed carbon through citrate secretion.

In summary, we have characterized the expression of *VuMATE1* in response to Fe deficiency. We conclude that this gene is not involved with citrate secretion from roots during Fe deficiency and nor is it involved with Fe translocation from the roots to the shoots. The role of *VuMATE1* in Al tolerance is controlled by elements in the promoter which respond to Al stress, via unknown pathways, to increase expression in root apices. The loss or gain of specific physiological functions in response to environmental selection pressures can occur via *cis* mutations that modify the level and distribution of gene expression.

## Author Contributions

JY and SZ conceived the study. ML, JX, HL, and WF performed the experiments and carried out the analysis. ML and JY designed the experiments and wrote the manuscript. All authors read and approved the final manuscript.

## Conflict of Interest Statement

The authors declare that the research was conducted in the absence of any commercial or financial relationships that could be construed as a potential conflict of interest.
