# Supplementary material for: Characterization of VuMATE1 Expression in Response to Iron Nutrition and Aluminum Stress Reveals Adaptation of Rice Bean (Vigna umbellata) to Acid Soils through Cis Regulation
Source: Front Plant Sci. 2016 Apr 19;7:511. doi: 10.3389/fpls.2016.00511 (PMC4835453; doi:10.3389/fpls.2016.00511)
Supplement: Supplementary file 1 [file Data_Sheet_1.DOC]

The following Supporting Information is available for this article:

Table S1 Primer sequences used in this study.

| Name | Application | Primer sequence (5'→3') |
| --- | --- | --- |
| GSP1 | Promoter isolation | GAGTCCTCCAAAGTATTGTGCTTATGT |
| GSP2 |  | TTGTGCGAGCCTTACTGCCTTATGT |
| TSS For | Identification of TSS | ACCAAAAAGATTCTTGTCGTT |
| TSS Rev |  | GGGAAAGGCAATCCCTAATA |
| -1720 bp Forward | GUS or GFP transgenic reporter lines | *Kpn*I GGGGTACCTTTTGGTCTTCAGTTAACAGAGTAAG |
| -1720 bp Reverse |  | *Nco*I CATGCCATGGTAATGAATTTTGATGAACTCACAGC |
| -192 bp Forward |  | *Kpn*I GGGGTACCGTTCCAACTGTGAACAAGACAAAAC |
| -574 bp Forward |  | *Kpn*I GGGGTACCGTGTTTGCTGTAATCTCACA |
| -1228 bp Forward |  | *Kpn*I GGGGTACCTAAGGCTCGCACAAGCTTCATTCTC |
| *VuMATE1* For | RT-PCR | CCTCTTGTGAGCATCACCAC |
| *VuMATE1* Rev |  | CGCGTAAGAGAGGTTTTGCT |
| *VuIRT1* For |  | GCATAATCGGTGTGACGCTA |
| *VuIRT1* Rev |  | CACGATAACGCACAAGCTGT |
| *VuMATE1* For | qRT-PCR | GCCAGAAGATCAGATGCT |
| *VuMATE1* Rev |  | GTTGTCCGTTGTGGCATT |
| *VuIRT1* For |  | GTGCTTCCCGATTCCTTTGAC |
| *VuIRT1* Rev |  | CCAAAGAATCCACCATCATCG |
| *18S rRNA* For |  | ATGATAACTCGACGGATCGC |
| *18S rRNA* Rev |  | CTTGGATGTGGTAGCCGTTT |
| VuMATE1p*::VuMATE1*  For | Complementation assay | *Bgl*II GAAGATCTATGGAAGAGAATGGTAGTTCCAATGA |
| VuMATE1p*::VuMATE1* Rev |  | *Bgl*II GAAGATCTAGCCAATGAACAACCTCTGAGATAAC |

**Figure S1** *VuMATE1* promoter structure analysis.

*VuMATE1* promoter structure analysis. (A) Schematic presentation of *VuMATE1* promoter structure. White boxes before ATG stand for the 5’ untranslated regions. Vertical black arrows represent positions of transcriptional (A, +1) and translational start site (ATG, +281). Horizontal red arrows indicate primer positions of 5’ deleted promoter fragments for construction of transgenic reporter lines. (B) Image of agarose gel showing 5’-RACE PCR products. M, size markers.

+1

**A**

+281

A

ATG

35/30 bp

-

192 bp

-

579 bp

-

1228 bp

-

1720 bp

-

TATAA

**192 bp::*GUS* For**

**-**

**579 bp::*GUS* For**

**-**

**1228 bp::*GUS* For**

**-**

**1720 bp::*GUS* For**

**-**

**1720 bp::*GUS* Rev**

**-**


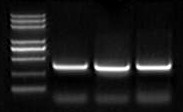


Clones

1

2

3

**B**

M

**500 bp**

**750 bp**

**100 bp**

**250 bp**

**Fig. S1**

**Figure S2** *In vivo* analysis of *VuMATE1* expression pattern and tissue localization in the transgenic Arabidopsis carrying VuMATE1p*::GFP*.

Ten-day-old seedlings of transgenic *Arabidopsis* plants harboring VuMATE1p*::GFP* were treated with Al (1 µM activity) (A and B) or not (A and C) for 9 h. GFP signal was detected by a laser-scanning confocal microscope. (A) and (B) root apex; (C) and (D) mature root zone. Bars, 100 µm.

**
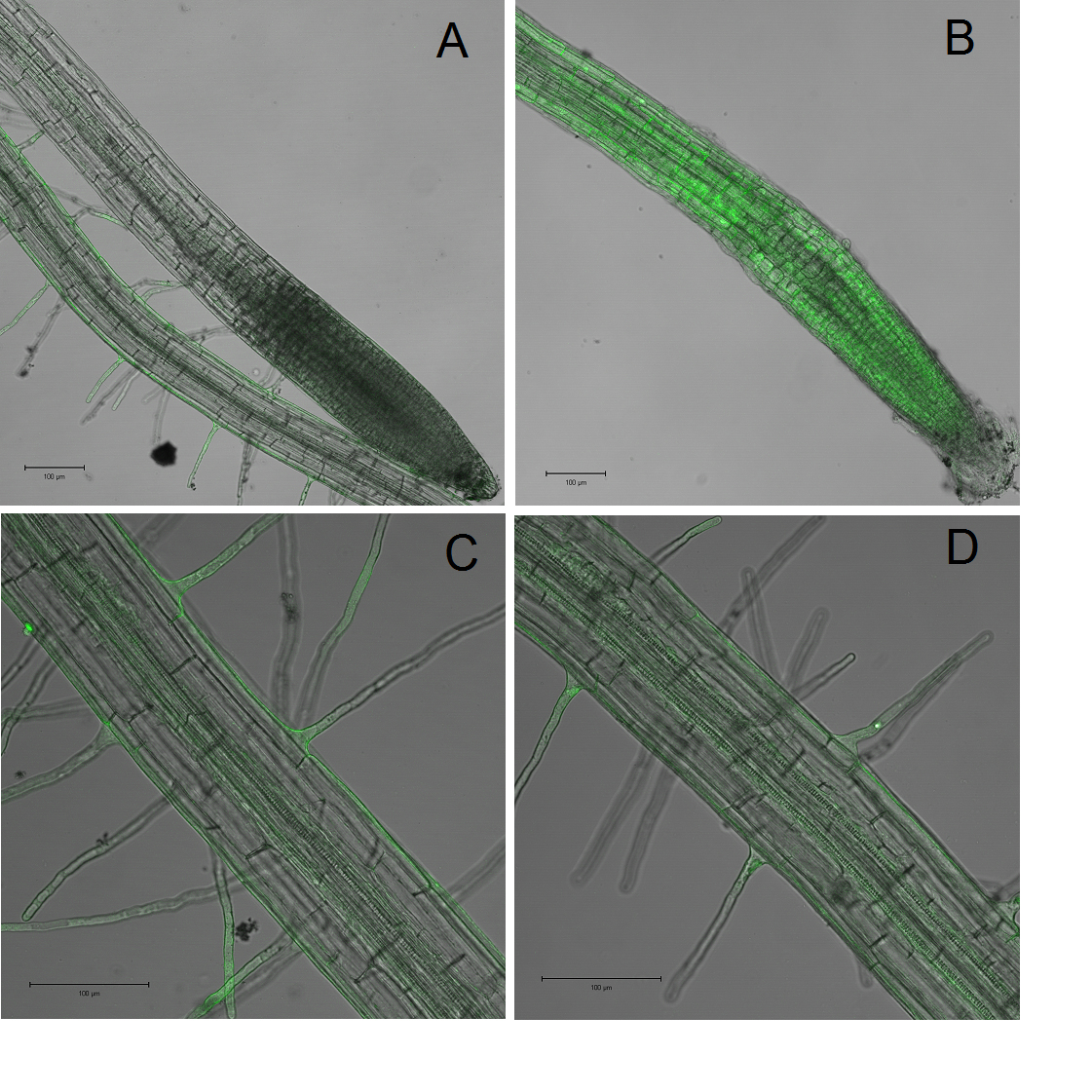
**

**Fig. S2**

**Figure S3** Fe-deficiency phenotype of rice bean leaves.

Photograph of rice bean plants grown in 1/5 Hoagland nutrient solution with (+Fe) or without (-Fe) 20 µM Fe for 12 d.

**Fig. S3**

**Figure S4** Root ferric precipitation in WT, *frd3-1*, and two complemented lines (line1 and line2).

Perls blue staining indicative of root ferric precipitation in WT, *frd3-1*, and two complemented lines (line1 and line2). Seeds were germinated and grown on Fe-sufficient medium for 3 weeks. Bars, 100 µm.

**Fig. S4**
